# Supplementary figures and images for: Expression Profiles of ID and E2A in Ovarian Cancer and Suppression of Ovarian Cancer by the E2A Isoform E47
Source: Cancers (Basel). 2022 Jun 12;14(12):2903. doi: 10.3390/cancers14122903 (PMC9221321; doi:10.3390/cancers14122903)

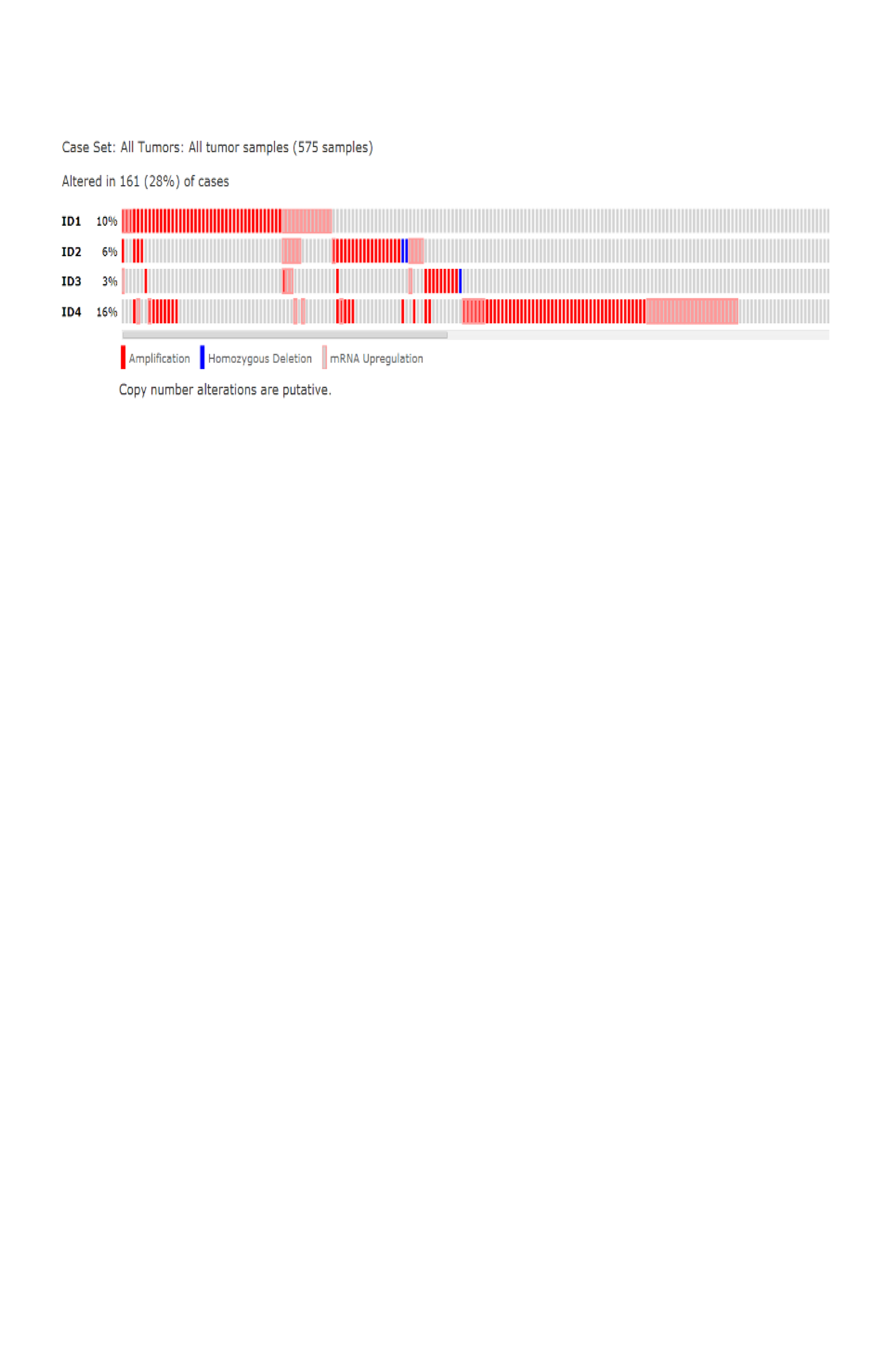

Supplement: Supplementary file 1 [file cancers-14-02903-s001.zip › Supplemenatal figure S1.tif]

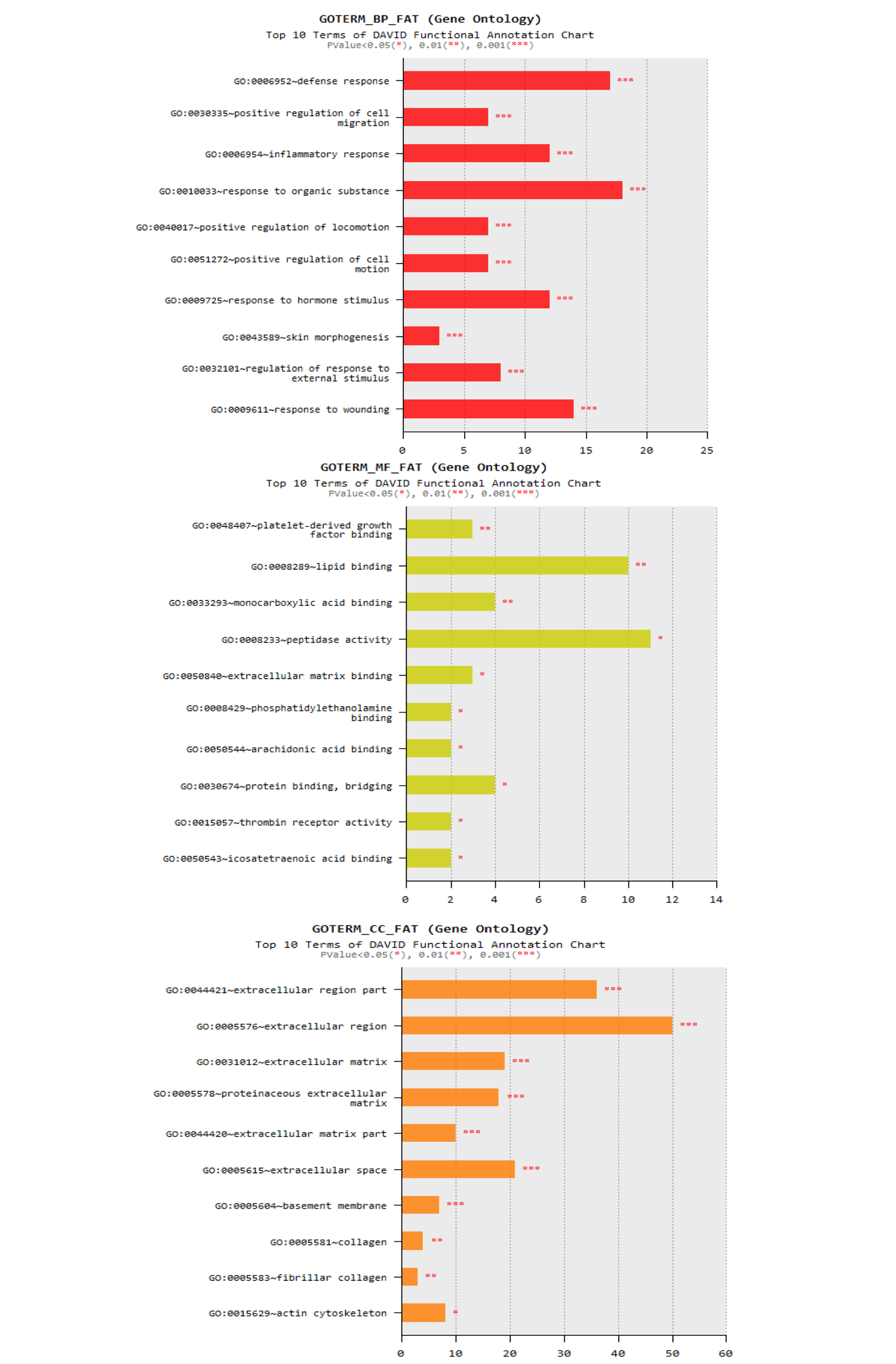

Supplement: Supplementary file 1 [file cancers-14-02903-s001.zip › Supplemenatal figure S2.tif]

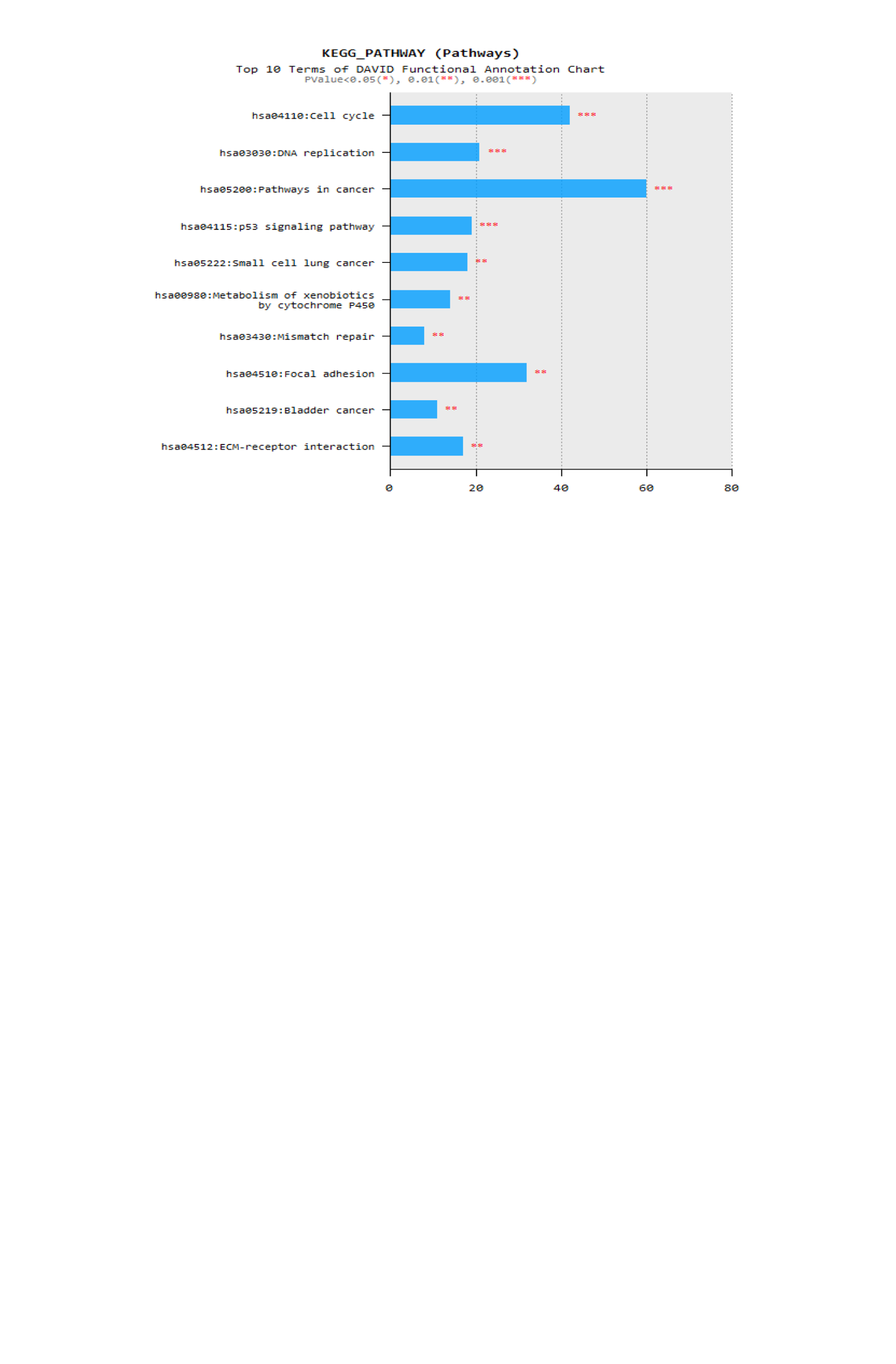

Supplement: Supplementary file 1 [file cancers-14-02903-s001.zip › Supplemenatal figure S3.tif]
